# Antimicrobial-Drug Prescription in Ambulatory Care Settings, United States, 1992–2000

**DOI:** 10.3201/eid0904.020268

**Published:** 2003-04

**Authors:** Linda F. McCaig, Richard E. Besser, James M. Hughes

**Affiliations:** * Centers for Disease Control and Prevention, Atlanta, Georgia, USA

**Keywords:** antimicrobial drugs, prescribing, antimicrobial resistance, physician offices, emergency departments, outpatient departments, research

## Abstract

During the 1990s, as antimicrobial resistance increased among pneumococci, many organizations promoted appropriate antimicrobial use to combat resistance. We analyzed data from the National Ambulatory Medical Care Survey, an annual sample survey of visits to office-based physicians, and the National Hospital Ambulatory Medical Care Survey, an annual sample survey of visits to hospital emergency and outpatient departments, to describe trends in antimicrobial prescribing from 1992 to 2000 in the United States. Approximately 1,100–1,900 physicians reported data from 21,000–37,000 visits; 200–300 outpatient departments reported data for 28,000–35,000 visits; ~400 emergency departments reported data for 21,000–36,000 visits each year. In that period, the population- and visit-based antimicrobial prescribing rates in ambulatory care settings decreased by 23% and 25%, respectively, driven largely by a decrease in prescribing by office-based physicians. Antimicrobial prescribing rates changed as follows: amoxicillin and ampicillin, –43%; cephalosporins, –28%; erythromycin, –76%; azithromycin and clarithromycin, +388%; quinolones, +78%; and amoxicillin/clavulanate, +72%. This increasing use of azithromycin, clarithromycin, and quinolones warrants concern as macrolide- and fluoroquinolone-resistant pneumococci are increasing.

With the emergence of antimicrobial resistance (*1*–*7*), the use of antimicrobial drugs has increased in both inpatient (*8*) and outpatient settings (*9*,*10*). From 1995 through 1998, the overall proportion of isolates of *Streptococcus* *pneumoniae*, a community-acquired pathogen, that were resistant to three or more antimicrobial drug classes rose substantially (*11*), and high rates of antimicrobial use for upper respiratory tract infections are believed to be a major factor responsible for this increase. Although the overall antimicrobial prescribing rate by office-based physicians in the United States did not change from 1980 through 1992, the rate for children rose by 48% (*12*), and in 1992, antimicrobial agents were prescribed second in frequency behind cardiovascular-renal drugs in physicians’ offices (*13*). Moreover, in the early 1990s, a sizable proportion of antibiotic prescriptions provided by office-based physicians to both children and adults were for colds, upper respiratory tract infections, and bronchitis, for which these drugs have little or no benefit (*14*,*15*).

During the 1990s, many organizations (e.g., the Centers for Disease Control and Prevention [CDC], American Academy of Pediatrics, American Academy of Family Practice, American Society of Microbiology, and Alliance for the Prudent Use of Antibiotics), conducted campaigns to promote appropriate antimicrobial use (*16*,*17*), defined by CDC as use that maximizes therapeutic impact while minimizing toxicity and the development of resistance. As a result of these and other efforts and increased media attention to the problem of antimicrobial resistance, antimicrobial prescribing for children seen in physician offices with respiratory infections decreased from 1989 through 2000 (*18*).

The objective of this study was to describe trends in antimicrobial prescribing at visits to office-based physicians, hospital outpatient departments, and hospital emergency departments in the United States. The results are based on a secondary data analysis using the 1992–2000 National Ambulatory Medical Care Survey (NAMCS) and National Hospital Ambulatory Medical Care Survey (NHAMCS).

## Methods

### Sample Design

NAMCS is a probability sample survey of office-based physicians in the United States conducted by CDC’s National Center for Health Statistics. The U.S. Bureau of the Census has been responsible for field operations and data collection since NAMCS became an annual survey in 1989. A report describing sample design, sampling variance, and estimation procedures of the NAMCS has been published (*19*). NAMCS uses a three-stage probability sampling procedure. The first stage contains 112 geographic primary sampling units. The second stage consists of a probability sample of practicing nonfederally employed physicians (excluding those in the specialties of anesthesiology, radiology, and pathology) selected from the master files maintained by the American Medical Association and the American Osteopathic Association. Physicians selected to participate in NAMCS during a particular calendar year are not eligible to be selected again for at least another 3 years. The third stage involves selecting patient visits to the sample physicians during a randomly assigned 1-week reporting period in that year.

NHAMCS is an annual probability sample survey of hospital outpatient departments and emergency departments in the United States, first conducted in 1992 by CDC’s National Center for Health Statistics. The U.S. Census Bureau is responsible for field operations and data collection. A published report describes the plan and operation of NHAMCS (*20*). NHAMCS uses a four-stage probability sampling procedure. The first-stage sample contains the same 112 geographic primary sampling units as NAMCS. The second stage consists of a probability sample of nonfederal, short-stay or general hospitals with emergency departments, outpatient departments, or both, within the sampled primary sampling units. Hospitals are selected from a publicly available database of all hospitals in the United States. The third stage involves selecting emergency service areas within emergency departments and clinics within outpatient departments. Clinics are classified into six groups: general medicine, including internal medicine; surgery; pediatrics; obstetrics/gynecology; substance abuse; and other, which includes clinics such as psychiatry and neurology. Clinics where only ancillary services are provided, such as radiology, physical therapy, and nutrition, are excluded. The fourth stage consists of sampling patient visits within emergency departments or clinics during a randomly assigned 4-week reporting period in that year.

### Response Rates and Sample Size

From 1992 through 2000, the response rates ranged from 63% to 73% for NAMCS, 86% to 91% for NHAMCS outpatient departments, and 93% to 97% for NHAMCS emergency departments. The NAMCS response rate was defined as the number of eligible physicians who completed the survey plus the number of eligible physicians who saw no patients during the study period, divided by the sum of the numerator and the number of physicians who refused to participate. The NHAMCS response rate was defined as the number of completed cases divided by the sum of the numerator plus the number of case-patients who refused. For each year of the study, the number of participating NAMCS physicians ranged from 1,100 to 1,900, the number of NHAMCS outpatient departments, from 211 to 283, and NHAMCS emergency departments, from 375 to 425. The number of patient record forms completed each year for NAMCS ranged from 21,000 to 37,000, for outpatient departments, from 28,000–35,000, and for emergency departments, from 21,000–36,000. The number of antimicrobial patient record forms completed each year for NAMCS ranged from 2,000 to 4,200; for NHAMCS outpatient departments, 2,800–3,500; and for NHAMCS emergency departments, 3,700–6,600.

### Data Collection and Coding

The same patient record form is used for both the physician’s office and outpatient department settings, whereas the emergency department form differs slightly to reflect the uniqueness of that setting. The form contains information about the visit, such as patient’s date of birth and medications prescribed. Physician specialty was recorded for NAMCS during a personal interview with the physician. Physicians and hospital staff were instructed to record all new or continued medications ordered, supplied, or administered at the visit, including prescription and nonprescription preparations, immunizations, desensitizing agents, and anesthetics. From 1989 through 1994, up to five medications were recorded per visit, and from 1995 through 2000, up to six medications were listed per visit. Drugs were coded according to a classification system developed at the National Center for Health Statistics. A report describing the method and instruments used to collect and process drug information has been published (*21*). For this analysis, five drugs were assessed per visit. Since data on the route of administration were not collected, an attempt was made to delete topical preparations by reviewing trade names and excluding those intended for topical use (*22*–*25*). For this article, antimicrobial drugs were defined as drugs belonging to the following groups: quinolones (including nalidixic acid); azithromycin and clarithromycin; erythromycin; amoxicillin and ampicillin; amoxicillin/clavulanate; other penicillins; cephalosporins; trimethoprim-sulfamethoxazole; and tetracyclines.

### Rate Definitions

Two types of antimicrobial drug use rates were used in the analysis. The population-based rate was defined as the annual number of antimicrobial drugs recorded in the three ambulatory care settings divided by the civilian noninstitutional population of the United States. The population-based rate accounts for any changes that may have resulted in a patient being less likely to have visited an ambulatory care setting (e.g., an increase in telephone advice, education from a healthcare provider, or changes in insurance status). The visit-based rate was defined as the annual number of antimicrobial drugs recorded in the three ambulatory care settings divided by the annual number of ambulatory care visits in the United States. The visit-based rate reflects changes in prescribing behavior once a visit has occurred.

### Statistical Analysis

Data from NAMCS and NHAMCS samples were weighted to produce national estimates. From 1992 through 1994, NAMCS weight included three components: selection probability, nonresponse adjustment, and physician-population weighting ratio adjustment. In 1995, a fourth component, weight smoothing, was added. NHAMCS weight includes three components: selection probability, nonresponse adjustment, and ratio adjustment to fixed totals. SUDAAN statistical software was used for all statistical analyses (*26*). The standard errors used to calculate the 95% confidence intervals (CI) around the estimates took into account the complex sample designs of NAMCS and NHAMCS. All estimates in this analysis had <30% relative standard error (i.e., the standard error divided by the estimate expressed as a percentage of the estimate) and were based on 30 cases or more in the sample data. Significance of trends was based on a weighted least-squares regression analysis at the 0.01 level of confidence (*27*).

## Results

**Figure 1:**
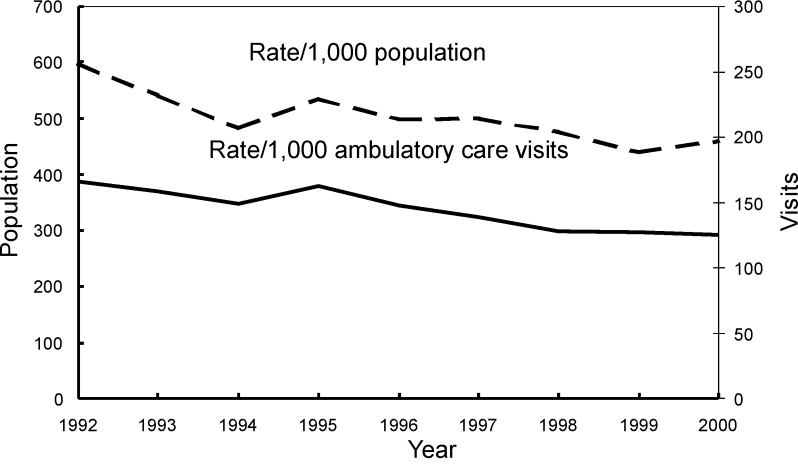
Trends in annual antimicrobial prescribing rates—United States, 1992–2000. Note: all trends shown are significant (p<0.001).

**Figure 2:**
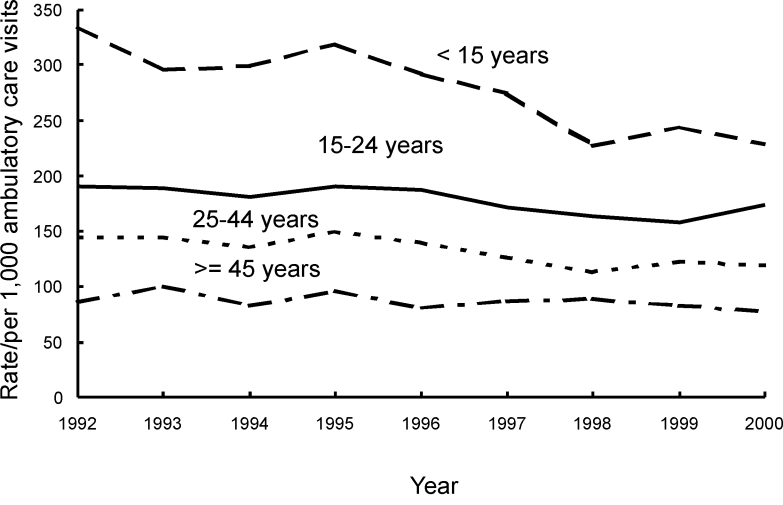
Trends in annual antimicrobial prescribing rates by age—United States, 1992–2000. Note: trend for visits by patients <15 years of age, p<0.001; for visits by patients 15–24 years, p=0.007; for visits by patients 25–44 years, p<0.001.

**Figure 3:**
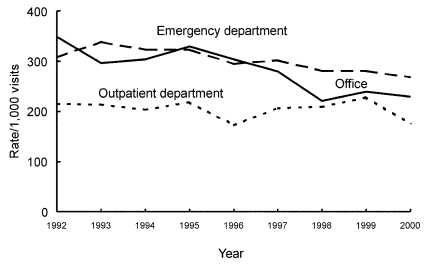
Trends in annual antimicrobial prescribing rates for persons <15 years of age by setting—United States, 1992–2000. Note: trend for office setting and emergency departments, p<0.001.

**Figure 4:**
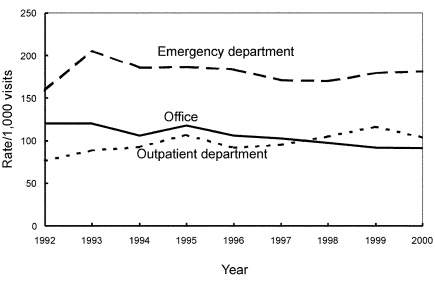
Trends in annual antimicrobial prescribing rates for persons >15 years of age by setting—United States, 1992–2000. Note: trend for office setting, p<0.001; trend for outpatient departments, p=0.002.

From 1992 through 2000, the number of antimicrobial drug prescriptions in ambulatory care settings in the United States declined from 151 million (95% CI 132 to 169) to 126 million (95% CI 112 to 141), while the number of visits rose from 908 million (95% CI 842 to 975) to 1.0 billion (95% CI 0.9 to 1.1). The annual population-based rate of antimicrobial drug use decreased by 23% (from 599 [95% CI 524 to 673] antimicrobial drug prescriptions per 1,000 persons to 461[95% CI 409 to 513]) (p<0.001), and the annual visit-based rate of antimicrobial drug use declined by 25% (from 166 [95% CI 152 to 179] antimicrobial drug prescriptions per 1,000 visits to 125 [95% CI 116 to 133]) (p<0.001) during the study period (Figure 1). All subsequent rates shown are visit-based rates. The antimicrobial prescribing rate at ambulatory care visits decreased in persons <15 years of age (–32%; p<0.001), 15–24 years (–9%; p=0.007), and 25–44 years of age (–17%; p<0.001). No trend was found among persons >45 years (p=0.03) (Figure 2). For children <15 years of age, antimicrobial prescribing rates decreased by 34% in physicians’ offices (p<0.001) and by 13% in emergency departments (p<0.001), but no trend was observed in the prescribing rates in outpatient departments (p=0.17) (Figure 3). The physician’s office was the only ambulatory care setting which experienced a decline in antimicrobial prescribing rates for persons >15 years (–24%; p<0.001), while an increasing trend was seen in outpatient departments (+35%; [is a p missing?]=0.002), and no change was observed in emergency departments (Figure 4). For visits to physician offices, antimicrobial prescribing rates decreased for general and orthopedic surgeons (–45%; p<0.001), general and family practitioners (–34%; p<0.001), pediatricians (–33%; p<0.001), and dermatologists (–4%; p=0.006) (Table 1).

**Table 1:** Trends in annual antimicrobial drug prescribing rates at physicians’ offices by specialty—United States, 1992–2000

Physician specialty	No. of antimicrobial drug prescriptions/1,000 visits^a^									% change since 1992
	1992	1993	1994	1995	1996	1997	1998	1999	2000	
Pediatrics	353 (310, 397)	325 (276,374)	302 (255,349)	344 (304,384)	340 (291,389)	299 (262,336)	218 (182,253)	258 (202,314)	235 (208,263)	–33^b^
General/Family practice	265 (232,298)	226 (199,254)	241 (216,267)	231 (204,258)	201 (178,225)	207 (181,234)	187 (164,209)	188 (160,216)	176 (148,204)	–34^b^
Otolaryngology	182 (141,223)	218 (177,259)	181 (146,217)	197 (153,241)	179 (147,210)	189 (122,256)	169 (135,203)	162 (97,227)	166 (128,205)	–8
Internal medicine	139 (114,165)	147 (117,178)	143 (111,174)	162 (137,187)	147 (114,180)	123 (97,149)	142 (122,162)	138 (104,173)	116 (95,136)	–17
Dermatology	138 (110,167)	149 (124,173)	140 (114,166)	134 (107,161)	116 (97,136)	106 (75,137)	112 (83,141)	92 (70,114)	133 (110,157)	–4^b^
Urology	118 (90, 145)	129 (100,158)	144 (117,172)	158 (120,196)	122 (85,159)	153 (108,199)	108 (84,133)	131 (89,172)	148 (123,172)	+26
General/Orthopedic surgery	40 (26,54)	39 (26,51)	30 (18,42)	30 (20,40)	39 (24,54)	44 (24,64)	14 (7,21)	28 (12,44)	22 (14,30)	–45^b^
All others	39 (28,49)	51 (34,68)	40 (31,49)	50 (32,69)	39 (26,51)	37 (28,47)	42 (30,53)	30 (22,38)	36 (27,45)	–6

^a^95% confidence interval. ^b^Trend is significant (p<.01).

**Figure 5:**
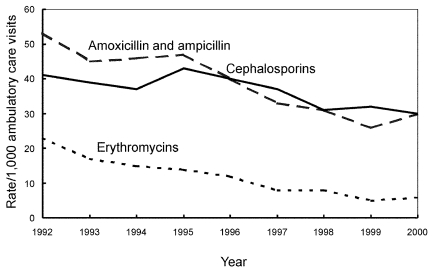
Trends in decreasing annual antimicrobial prescribing rates by drug class—United States, 1992–2000. Note: all trends shown are significant (p<0.001).

**Figure 6:**
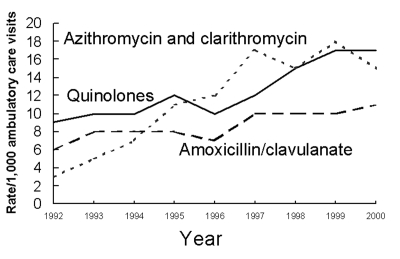
Trends in increasing annual antimicrobial prescribing rates by drug class—United States, 1992–2000. Note: trend for amoxicillin/clavulanate prescribing among children<15 years of age, p=0.004; for quinolones among persons >15 years, p<0.001; for azithromycin and clarithromycin among all ages, p<0.001.

During the study period, the antimicrobial prescribing rate at all ambulatory care visits declined for amoxicillin and ampicillin (–43%;p<0.001), cephalosporins (–28%; p<0.001), and erythromycin (–76%; p<0.001) (Figure 5); the prescribing rate rose for azithromycin and clarithromycin (+388%; p<0.001), quinolones among persons >15 years (+78%; p<0.001), and amoxicillin/clavulanate among children <15 years (+72%; p=0.004) (Figure 6). Decreasing trends were also found for other penicillins (p<0.001), tetracyclines (p<0.001), and trimethoprim-sulfamethoxazole (p=0.009) (data not shown). Table 2 shows the rank order of the nine drug classes examined in 1992 compared with their order in 2000.

**Table 2:** Rank order of antimicrobial drug classes in ambulatory care settings, 1992 and 2000

Antimicrobial drug class	1992	2000
Amoxicillin and ampicillin^a^	1	1
Cephalosporins	2	2
Erythromycin	3	8
Tetracyclines	4	7
Other penicillins	5	9
Trimethprim-sulfamethoxazole	6	6
Quniolones	7	4
Amoxicillin/clavulanate	8	5
Azithromycin and clarithromycin^b^	9	3

^a^Excludes amoxicillin/clavulanate. ^b^Only clarithromycin was in the National Ambulatory Medical Care Survey and National Hospital Ambulatory Medical Care Survey drug databases in 1992.

## Discussion

Our study found decreasing trends in both the population- and visit-based antimicrobial prescribing rates in ambulatory care settings from 1992 through 2000. The population-based prescribing rate provides the number of antimicrobial drugs used per person in the United States; we used this rate to assess changes over time that may be attributed to variations in visiting an ambulatory care setting. Declining population-based antimicrobial prescribing rates may be a result of several factors: a decrease in visits which, for example, may be due to a decrease in the incidence of a disease or changes in the patient’s health insurance coverage; a decrease in prescribing, which may be the result of an increased understanding by the patient and/or healthcare provider of the impact of antimicrobial use, or both. Declining visit-based antimicrobial prescribing rates only reflect a change in prescribing behavior occurring at ambulatory care visits.

The decreasing trends in the antimicrobial prescribing rate found in this study for both children and adults seen in physicians’ offices from 1992 through 2000 contrast with findings of a previous report that examined NAMCS data from 1980 through 1992. That report showed an increasing trend in antimicrobial prescribing for children and no trends for the older age groups (*12*). Although NAMCS data for children have been published previously in a slightly different format (*18*), showing the prescribing rates in all three settings is important to understanding practice patterns in ambulatory care. The findings suggest that efforts to promote appropriate antimicrobial use in physicians’ offices may be effective.

Increasing rates of use were observed for some of the new, more expensive, broad-spectrum antimicrobial agents, such as azithromycin and clarithromycin, quinolones, and amoxicillin/clavulanate. The large increase in the use of azithromycin and clarithromycin may be partially explained by the fact that clarithromycin was first mentioned in NAMCS and NHAMCS in 1992 and azithromycin in 1993. While these agents have been recommended for use in some patients with community-acquired pneumonia (*28*), cases of pneumonia are unlikely to account for this dramatic increase in their use. Fluoroquinolones and newer macrolides (azithromycin and clarithromyicn) are rarely indicated as first-line therapy for other respiratory infections (*29*,*30*). The decrease in the use of amoxicillin and ampicillin could be a consequence of the 46% decrease in visits to physician offices for otitis media from 1989 through 2000 (*18*).

Antimicrobial use, whether appropriate or inappropriate, promotes antimicrobial resistance. The increasing use of azithromycin, clarithromycin, and fluoroquinolones warrants concern in light of the importance of these agents in the treatment of patients hospitalized with pneumonia, and the rise in macrolide- and fluoroquinolone-resistant pneumococci in many parts of the world (*11*,*31*–*35*). Making certain that the increasing use of these agents is clinically appropriate is important. While most efforts to date promoting appropriate antibiotic use have focused on reducing the use of antimicrobial agents for viral infections, future efforts should be directed towards ensuring that when antimicrobial agents are indicated, first-line or targeted therapy is employed.

Decreasing trends in antimicrobial drug prescribing rates were found for office visits to pediatricians, general and family practitioners, dermatologists, and general and orthopedic surgeons. Interventions may need to be tailored differently to different settings (e.g., physician’s office versus outpatient department versus emergency department) and physician specialty groups. In 2000, the American College of Physicians-American Society of Internal Medicine (ACP-ASIM) designated antimicrobial resistance as a focus for their continuing medical education conferences. The ACP-ASIM, together with CDC and the American Academy of Family Physicians and the Infectious Diseases Society of America, has published principles for appropriate prescribing for upper respiratory infections in adults (*29*). These principles will form the scientific basis for new campaigns to improve prescribing by clinicians who treat adults. Future analyses of NAMCS and NHAMCS data will show whether these activities result in changes in prescribing behavior similar to those seen for children.

The major limitation of our study is that the appropriateness of an antimicrobial prescription cannot be assessed in most instances because diagnosis is not linked to a particular drug. Patient visits in NAMCS or NHAMCS do not include telephone contacts; therefore, we could not determine whether a shift to telephone prescribing for antimicrobial agents occurred. However, we could assess whether prescribing had made a transition from physicians’ offices to emergency departments or outpatient departments. A shift to other healthcare settings (at least for children <15 years of age) did not appear to occur because a decreasing trend was also found in emergency departments in addition to physicians’ offices, and outpatient departments did not show a trend. However, for adults, antimicrobial drug prescribing declined in physicians’ offices, remained the same in emergency departments, and rose in outpatient departments, suggesting that a change in setting could have occurred.

The dynamics that influence antimicrobial prescribing are complex. In recent years, physicians have been receiving messages about the appropriate use of antimicrobial drugs from the medical literature, the media, health insurance companies, key opinion leaders, alternative medicine leaders, and patients (*36*). These messages appear to have been absorbed to some extent, as evidenced by the results shown in this article and the decline in antimicrobial prescribing in children seen in physicians’ offices (*18*). However, the increasing use of azithromycin, clarithromycin, and quinolones evokes concern and requires additional study to determine their appropriateness. New efforts must be made to promote targeted agents as first-line therapy.
