# Correction Vol. 9, No. 4

**DOI:** 10.3201/eid0905.020268

**Published:** 2003-05

In the article, “Antimicrobial Drug Prescriptions in Ambulatory Care Settings, United States, 1992–2000” by Linda F. McCaig et al, errors occurred on pages 432, 434, and 446. On page 432, the correct affiliations are as follows: Linda F. McCaig, National Center for Health Statistics, Centers for Disease Control and Prevention (CDC), Hyattsville, Maryland, USA; Richard E. Besser and James M. Hughes, National Center for Infectious Diseases, CDC, Atlanta, Georgia, USA. In the abstract, the change in antimicrobial prescribing rate for amoxicillin/clavulanate is +69%. On page 434, second paragraph, results section, the correct first sentence appears below:

During the study period, the antimicrobial prescribing rate at all ambulatory care visits declined for amoxicillin and ampicillin (-43%; p<0.001), cephalosporins (-28%; p<0.001), and erythromycin (‑76%; p<0.001) (Figure 5); the prescribing rate rose for azithromycin and clarithromycin (+388%; p<0.001), quinolones among persons >15 years (+78%; p<0.001), and amoxicillin/clavulanate (+69%; p=0.004) (Figure 6).

On page 436, the correct caption to Figure 6 appears below:

Trends in increasing annual antimicrobial prescribing rates by drug class—United States, 1992–2000. Note: trend for amoxicillin/clavulanate p<0.001; for quinolones among persons >15 years, p<0.001; for azithromycin and clarithromycin among all ages, p<0.001.

The corrected article appears online at http://www.cdc.gov/ncidod/EID/vol9no4/02-0268.htm.

We regret any confusion these errors may have caused.

